# Sovereignty-as-a-service: How big tech companies co-opt and redefine digital sovereignty

**DOI:** 10.1177/01634437251395003

**Published:** 2025-11-11

**Authors:** Rafael Grohmann, Alexandre Costa Barbosa

**Affiliations:** 1 University of Toronto, ON, Canada; 2 University of Arts Berlin, Germany

**Keywords:** digital sovereignty, discourse, sovereignty-as-a-service, big tech sovereignty

## Abstract

This article introduces the concept of sovereignty-as-a-service to describe how Big Tech companies, specifically Microsoft, Amazon, and Google/Alphabet, are strategically redefining digital sovereignty through their programs of cloud infrastructure. Drawing on critical discourse analysis of official materials released between 2022 and 2023, we show how these companies respond to regulatory pressures, particularly in Europe, by offering modular and branded solutions that frame sovereignty as a technical, legal, and infrastructural matter. Rather than sovereignty being exercised over platforms, it is now provisioned by them, on their terms. We argue that sovereignty-as-a-service constitutes a form of discursive capture that empties the concept, aligning it with the ideological legacy of the Californian Ideology. In this reframing, digital sovereignty becomes a service to be purchased, configured, and optimized through proprietary platforms. By conceptualizing sovereignty as a site of contested meanings open to appropriation, this article contributes to critical debates on digital sovereignty and technology governance.

## Introduction

Digital sovereignty has become one of the most contested concepts in internet policy and tech governance debates (Herlo et al., 2021). Once primarily associated with state control over territorial infrastructures, the term now circulates in corporate white papers, transnational institutes, think tanks, regulatory frameworks, and grassroots calls for sovereignty. In this discursive proliferation, the meanings of sovereignty have become deeply unstable, with struggles over meanings (Hall, 1980).

Over the past few years, major technology companies, particularly Microsoft, Amazon, and Alphabet/Google, have rebranded themselves as stewards of digital sovereignty. In response to mounting regulatory pressures, especially in Europe, these firms have launched programs that promise governments and organizations greater “control” over their data, infrastructures, and cloud operations. These initiatives, which we call “sovereignty-as-a-service,” means a strategic shift: sovereignty is no longer a matter of collective self-determination but a modular product delivered via cloud compliance, infrastructure management, and corporate-led governance frameworks. In other words, sovereignty becomes an empty signifier, one that Big Tech companies fill with infrastructural services, serving as a form of social responsibility whitewashing.

This essay argues that the discourse of “Big Tech sovereignty,” that is, Big Tech actively co-opting and controlling the meanings of sovereignty. constitutes an ideological operation that reconfigures political sovereignty into a commercial and commodified logic. Drawing on critical discourse analysis of the digital sovereignty programs launched by Microsoft, Amazon, and Google between 2022 and 2023, we examine how these companies frame sovereignty as a technical and infrastructural issue (Lehdonvirta, 2022; Plantin et al., 2018; Poell et al., 2021), reframing and delegitimizing its political, epistemic, and anti-colonial dimensions. Far from merely adapting to regulatory frameworks, these companies are actively reshaping the meaning of sovereignty to suit their infrastructural and geopolitical interests. This reconfiguration is not neutral.

As scholars of digital sovereignty have shown (Couture and Toupin, 2019; Pohle and Thiel, 2020; Rikap et al., 2024), the term carries conflicting meanings for different actors: states, individuals, social movements, and corporations. When Microsoft offers a cloud designed for “government sovereignty,” or when Google presents digital sovereignty as “a journey” that leads to its own services, these are not just corporate responses to external demands, they are strategic appropriations of a concept that historically belonged to political communities, not private firms.

Moreover, the framing of sovereignty-as-a-service is part of a broader tendency we identify as epistemic anti-sovereignty, a sustained attempt to maintain the U.S.-based control over global technological knowledge and infrastructure while appearing to localize and decentralize. This concept, grounded in the premises of epistemic sovereignty (Oliveira and Pinto, 2024), can be understood as a manifestation of tech and media imperialism (Albuquerque, 2024; Mirrlees, 2024), through the construction of intellectual monopolies (Rikap, 2024) and the epistemic co-optation of how digital sovereignty is meant to circulate across different spaces. In doing so, Big Tech recycles the ideological lineage of the “Californian Ideology” (Barbrook and Cameron, 1995; Marwick, 2017), in which libertarian promises of empowerment obscure the consolidation of platform power, renewing tech discourses in the context of “Silicon Valley dystopianism” (Karppi and Nieborg, 2021). Digital sovereignty, then, is at the center of ongoing discursive struggles over tech power.

This discursive power of tech companies constitutes an important facet of platform power itself vis-à-vis the state (Lehdonvirta, 2022; van Dijck et al., 2019), given that governments are the potential clients of these services. By selling “sovereign clouds,” states can maintain a discourse of “digital sovereignty” while in practice failing to achieve it. As corporations capture the agenda, pressure for regulatory frameworks and policies that genuinely address digital sovereignty, from both state and community perspectives, weakens, thereby reinforcing platform dependency (Grohmann, 2025), particularly in relation to infrastructures. By critiquing this discursive co-optation, this article calls on scholars, policymakers, and communities to engage in dialog on how to confront this situation.

## The many faces of digital sovereignty

Digital sovereignty is not a fixed or universally agreed-upon concept. Instead, it functions as a contested field of meaning, a site where multiple actors with conflicting agendas seek to define the terms of technological governance, whether data, platforms, artificial intelligence or other technologies. In 2019, Couture and Toupin published one of the foundational articles on the topic: “What does the notion of ‘sovereignty’ mean when referring to the digital?” (Couture and Toupin, 2019). There, they reveal the multiplicity and complexity of the term, showing that sovereignty in the digital realm cannot be reduced to any single dimension or actor, but can even be reclaimed by social movements, communities and workers (Couture et al., 2024).

However, dominant approaches to digital sovereignty generally fall into three main categories (Pohle and Thiel, 2020). The first concerns state control over digital infrastructures and the capacity to design and implement digital policies, often with an emphasis on cybersecurity. The second pertains to the broader digital economy, encompassing the role of national technology companies and the state in developing effective industrial and innovation policies. The third focuses on the individual or personal dimension of digital sovereignty, particularly the right to digital self-determination, user agency, and the ability to make informed decisions regarding personal data and algorithmic environments. Since the publication of these frameworks, the concept of digital sovereignty has gained increasing relevance in internet policy debates and has been mobilized in diverse and often conflicting ways by states, corporations, social movements, think tanks, scholars, communities, and policymakers around the world.

An alternative perspective approaches digital sovereignty through the lens of social movements. As Couture and Toupin (2019, p. 2315) observe, this understanding “is used to assert the autonomy of social movements through the collective (and sometimes individual) control of digital technologies and infrastructures, and particularly the power to develop and use tools,” often grounded in principles of free and open-source technologies. This perspective foregrounds critical questions about how social movements reclaim digital infrastructures from below and interact with institutions such as the state in the pursuit of digital sovereignty,

Taken together, these framings illustrate that digital sovereignty functions as a floating signifier, mobilized across multiple scales and in service of divergent political and economic agendas. It can evoke national self-sufficiency, liberal individualism, infrastructural autonomy, or collective resistance. These meanings also shift across geographic contexts: what digital sovereignty signifies in a European policy framework may differ substantially from how it is invoked in Latin America, Africa, or Asia. Rather than seeking to establish a fixed conceptual essence, our approach understands digital sovereignty as a discursive field marked by contestation among diverse actors and institutions.

This discursive plasticity is precisely what renders it so vulnerable to corporate capture. A brief look at Google Trends reflects this shifting landscape. Global interest in the term “digital sovereignty” strengthened between July and December 2022, the same period during which Amazon, Microsoft, and Alphabet/Google launched major initiatives focused on digital sovereignty in the European context. Beyond Europe, the term also gained traction in BRICS countries such as Brazil and India, suggesting that it is no longer confined to policy debates in the Global North. Instead, digital sovereignty has become a global discursive object, increasingly entangled in geopolitical struggles over tech debates.

## Methods

Through critical discourse analysis (CDA; Fairclough, 1992, 2003), this article examines the digital sovereignty programs launched by Google, Microsoft, and Amazon between 2022 and 2023. These companies were selected due to their combined control of over two-thirds of the global cloud computing market. CDA enables us to understand how specific textual features relate to broader discursive formations and political-economic contexts, in this case, how the term “sovereignty” is discursively mobilized and how this mobilization aligns with the strategic interests of major tech companies.

Data collection focused on official materials published on each company’s website, including program announcements, blog posts, and product descriptions. Our aim here was not to analyze the various discursive genres and formats employed by the companies, but rather to focus on their textual dimensions. Nevertheless, we observed that most of the materials displayed similar characteristics, generally taking the form of visually appealing websites created to present what are referred to as digital sovereignty programs. A thematic coding process was conducted, and following Fairclough (1992, 2003), we applied the concepts of utterance and discourse to identify the broader discursive formations, understood as ideologies and value systems, or ideological-discursive formations, to which each utterance was related.

Our analysis considered three core dimensions: (1) how sovereignty is defined or implied in each program; (2) the political and regulatory context in which the program was launched; and (3) the strategies deployed by each company—such as the specific products or services offered—under the banner of digital sovereignty. These materials were then interpreted in light of broader discourses and contested claims surrounding digital sovereignty in contemporary internet governance debates.

## What sovereignty means to big tech

All three companies—Google/Alphabet, Amazon, and Microsoft—launched their digital sovereignty programs between 2022 and 2023, with a clear focus on the European context. These initiatives emerged in response to recent regulatory developments and growing discourses around data governance and sovereignty in the region.

In late 2022, Amazon, through its cloud infrastructure division, Amazon Web Services, introduced the “Digital Sovereignty Pledge^1^.” The program was made available in multiple languages and promoted through local media outlets across different regions. For instance, in Brazil, an online news portal announced Amazon’s “commitment to digital sovereignty^2^.” According to the company, digital sovereignty is defined as “having control over digital assets,” and Amazon positions itself as enabling customers to meet sovereignty-related requirements. In this framing, sovereignty is effectively reduced to a question of asset management and consumer access to Amazon’s infrastructure.

Amazon invokes the concept of being “sovereign-by-design”—a term left undefined in official materials. This notion encompasses four main elements: (1) control over data location; (2) verifiable control over data access; (3) end-to-end encryption capabilities described in universal terms (“everything” and “everywhere”); and (4) cloud resilience, primarily tied to hardware-level security. These features are marketed as providing customers with enhanced control over infrastructure in compliance with existing legal frameworks, and are discursively framed as innovative and disruptive.

Microsoft was the first among the three companies to launch a digital sovereignty program, introducing Microsoft Cloud for Sovereignty in July 2022.^3^ The initiative is targeted primarily at governments and public sector clients seeking to invest in digital sovereignty. Here, sovereignty is again closely tied to cloud infrastructure and data governance, particularly through the concept of “data sovereignty.” According to Microsoft, “data sovereignty is the concept that data is under the control of the customer and governed by local law.” This definition aligns the company’s offering with national regulatory frameworks such as the General Data Protection Regulation (GDPR), positioning it as a partner in state compliance.

The central product associated with Microsoft’s program is the public cloud service Microsoft Azure. The company emphasizes that “every government has a unique view and requirements when addressing their sovereign needs,” and proposes to meet these needs through an “additional layer of policy and auditing capabilities” embedded in its cloud services. Microsoft’s framing suggests that, rather than developing autonomous digital sovereignty strategies, states can instead rely on Microsoft to make them “more sovereign,” thereby reinforcing the company’s infrastructural and discursive power in relation to state actors (Lehdonvirta, 2022).

Alphabet/Google was the last of the three companies to launch a sovereignty-focused initiative. In March 2023, it introduced the Digital Sovereignty Explorer, also designed with the European context in mind.^4^ Unlike Amazon and Microsoft, Google did not release new products but instead developed a tool to help customers identify “sovereignty solutions” related to data governance, operations, and software architecture, explicitly stating that sovereignty extends beyond data residency. After assessing client needs, the tool generates a report recommending appropriate Google Cloud services.

Google frames sovereignty as a service to be achieved through its ecosystem of tools, particularly Google Cloud and Google Workspace. The company describes digital sovereignty as “organizations maintaining control and autonomy as they develop their digital transformation and cloud strategies,” emphasizing features such as control, visibility, and transparency. Additionally, the program introduces a notion of software sovereignty tied to resilience “against disruptive geopolitical events.” In this framing, Google positions itself as a protector of customers’ interests, offering not just technical solutions but a form of geopolitical shielding that guarantees their digital sovereignty.

Despite their distinct strategies and framings, the digital sovereignty programs launched by Amazon, Microsoft, and Google between 2022 and 2023 converge in presenting sovereignty as a service offered by private infrastructures, rather than a political capacity developed by public institutions. Each company aligns its program with the regulatory pressures emerging from the European context, particularly in response to debates around data protection, AI regulation, and infrastructural autonomy. Amazon emphasizes customer-centric control over digital assets, Microsoft positions itself as a compliance partner for states, and Google offers sovereignty as an optimized configuration of its own cloud services. In all cases, sovereignty is disembedded from its historical and political roots and redefined as a configurable solution delivered through proprietary platforms.

While Amazon’s discourse emphasizes asset control and encryption, Microsoft frames sovereignty in terms of legal compliance and policy flexibility for governments. Google, by contrast, centers its narrative on digital transformation and infrastructural resilience, particularly in response to geopolitical disruptions. Across these cases, the logic is inverted: rather than states or communities exercising sovereignty over technologies, it is platforms that now grant the tools for others to be “sovereign,” on their terms. This sovereignty is ostensibly “localized,” but remains deeply dependent on the infrastructures and businesses of these global corporations. The Table 1 summarizes the main approaches of the digital sovereignty initiatives implemented by Big Tech between 2022 and 2023.

**Table 1.** Digital sovereignty programs by big tech companies.

Company	Program name	Launch date	Target audience	Core framing of sovereignty	Main features/tools
Amazon	Digital Sovereignty Pledge	Late 2022	Enterprise customers	Control over digital assets	Data location, access control, encryption, hardware-level security
Microsoft	Microsoft Cloud for Sovereignty	July 2022	Governments, public sector	Data sovereignty governed by local law	Azure public cloud, policy and auditing layers, GDPR compliance
Google	Digital Sovereignty Explorer	March 2023	Enterprise, governments	Sovereignty as digital transformation and resilience	Diagnostic tool, customized reports, geopolitical risk mitigation

Source: Authors’ compilation.

## Conclusion

The digital sovereignty programs of Amazon, Microsoft, and Google reveal a broader ideological shift in how platform companies engage with policy, political and academic concepts, co-opting according to their interests, as a response to the current regulatory frameworks. By reframing sovereignty as a service, these companies disembed the notion of sovereignty from its historical associations with collective self-determination, the role of State and local developments. In its place, they offer branded frameworks of “control,” “compliance,” and “resilience,” distributed through proprietary infrastructures.

This article has argued that this is not merely a response to regulatory pressures—particularly within the European Union, but a broader social synthesis of how Big Tech companies are seeking to update the Silicon Valley or Californian ideology by appropriating and hollowing out the meanings of key concepts emerging from civil society. This is what we call sovereignty-as-a-service. This is a form of discursive capture in which platform companies define the conditions under which others, whether individuals, corporations, or even governments, can be deemed “sovereign,” depending on their resources and infrastructural capacities. Rather than sovereignty being exercised over platforms, it is now granted by them, through tools and services that reproduce dependence on their infrastructures. Despite the variations of their “digital sovereignty programs,” all three companies share a fundamental move: they hollow out the political dimensions of sovereignty and reconstitute it as a marketable solution.

The originality of this analysis lies in demonstrating how sovereignty has become a discursive object of corporate appropriation, a floating signifier that platform companies fill with strategic meaning depending on region, audience, and political context. By tracing this process, we contribute to broader critical debates on platform power, Big Tech discourse, and digital sovereignty, approaching sovereignty not as something essentialized or homogeneous, but precisely as a concept under contestation and subject to capture. Ultimately, this reframing compels us to ask not just who locally controls the cloud, but who controls the meaning of sovereignty itself, and to what ends.

We do not intend here to analyze how these services are being purchased by governments around the world, but it is possible to state that this form of tech power, both discursive and infrastructural, is already producing effects. The Brazilian government, for instance, under President Lula, consistently promotes a discourse of sovereignty while simultaneously purchasing “sovereign” cloud services from Amazon (Dias, 2025). This illustrates how such discursive dissimulation can further reinforce platform power.

In addressing this issue, the authors also invite the academic community to consider how digital sovereignty can be reclaimed through other ways, like popular power, or a notion of popular digital sovereignty, and by different communities from below, as a way of confronting platform dependency. The Homeless Workers’ Movement in Brazil (MTST), for instance, has been advancing this debate from below (MTST, 2023; Salvagni et al., 2024).

## Notes

1. Available at: https://aws.amazon.com/pt/blogs/security/aws-digital-sovereignty-pledge-control-without-compromise/

2. Available at: https://www.nexojornal.com.br/extra/2022/11/28/Amazon-anuncia-compromisso-com-a-soberania-digital

3. Available at: https://www.microsoft.com/en-us/industry/sovereignty/cloud

4.
https://cloudsovereignty.withgoogle.com/
